# Supplementary material for: Distinct functional elements for outer-surface anti-interference and inner-wall ion gating of nanochannels
Source: Nat Commun. 2018 Nov 1;9:4557. doi: 10.1038/s41467-018-06873-z (PMC6212446; doi:10.1038/s41467-018-06873-z)
Supplement: Supplementary file 1 — Supplementary Information [file 41467_2018_6873_MOESM1_ESM.pdf]

Supplementary Information

Role of function-element at outer-surface for anti-interference and function-element at inner-wall for ion-gating of nanochannels

Gao et al

Table of the contents

Supplementary Methods

Supplementary Figures

Supplementary Figure 1 | Sketch map of metallic depositions

Supplementary Figure 2 | Linear scanning of EDS along nanochannels

Supplementary Figure 3 | The sectional view of the membrane after TOF-SIMS

Supplementary Figure 4 | Thickness and the depth of the deposited elements

Supplementary Figure 5 | Statistic of geometrical parameter of the membranes

Supplementary Figure 6 | Sketch map of the silane coupling reaction

Supplementary Figure 7 | The LSCM images of DNA-Cy5 along nanochannels

Supplementary Figure 8 | The I-V curves after the metallic depositions.

Supplementary Figure 9 | I-V curves of the membrane with pore size (55 ± 15 nm)

Supplementary Figure 10 | Sketch map of the DNA hybridization

Supplementary Figure 11 | The LSCM images of cpDNA-Cy5 along nanochannels

Supplementary Figure 12 | I-V curves of the membrane modified with ssw-DNA

Supplementary Figure 13 | Detection of ATP by using the nanochannels

Supplementary Figure 14 | EIS sketch and equivalent circuit

Supplementary Figure 15 | Gating efficiency of FE_{OS} and FE_{IW} by EIS

Supplementary Figure 16 | Comparison of DNA amount at different domains

Supplementary Figure 17 | I-V curves of the superficially functionalized membranes

Supplementary Figure 18 | The EIS spectra of the membranes in Fig. 5 and Fig. 6

Supplementary Figure 19 | Contact angle tests of the functionalized membranes

Supplementary Figure 20 | Zeta potential of PEI or PAA coating membrane

Supplementary Figure 21 | The chosen FE_{OS} with the anti-interference as Fig. 5

Supplementary Figure 22 | The chosen FE_{OS} with the anti-interference as Fig. 6

Supplementary Figure 23 | The radar map of anti-interference after 2 V driving

Supplementary Figure 24 | Analogical function divisions in a nuclear pore complex.

Supplementary Tables

Supplementary Table 1 | Names of used samples

Supplementary Table 2 | Abbreviations

Supplementary Table 3 | DNA sequences

Supplementary Discussion:

Supplementary Table 4 | Depth calculations by TOF-SIMS and LSCM

Supplementary Figure 25 | The profile of depth *vs.* absolute intensity for TOF-SIMS

Supplementary Methods

Materials

All oligonucleotides were synthesized and purified by Sango Biotech Co. Ltd. (Shanghai, China), and the sequences are shown in Table S2. NaCl, $MgCl_2$, Tris-(2-carboxyethyl) phosphine hydrochloride (TCEP) were obtained from Sigma Aldrich. Polyacrylic acid (PAA, MW=50, 000, 50 wt% aqueous solution) and Polyetherimide (PEI, MW=10,000, 99 wt% aqueous solution) were purchased from Macklin Co. Ltd. (Shanghai, China). (3-aminopropyl)triethoxysilane (APTES, MW=221.4) and Perfluorooctyltriethoxy silane (PTOS, MW=510.36) was purchased from Alfa Aesar Co. Ltd. (Shanghai, China). The Silole-SNBr is a water-soluble molecule in the nature of aggregation-induced emission (AIEgens) (the molecular formula were listed in the bottom of this section). The molecular weight of Silole-SNBr is 965.3. The $\lambda_{excitation}$ and $\lambda_{emission}$ are 466 nm and 580 nm measured by fluorescence spectrometer. AAO membranes were purchased from Pu-Yuan Nano Technology Co. Ltd. (Hefei, China). AAO membranes were prepared by classical anodic oxidation and gentle acidic etching treatment. The thickness of AAO membrane is 40 μm. All other chemicals are of analytical grade, and all chemicals were used without further purification. All solutions were prepared with ultrapure water (resistivity=18.2 MΩ·cm) from a Millipore system.

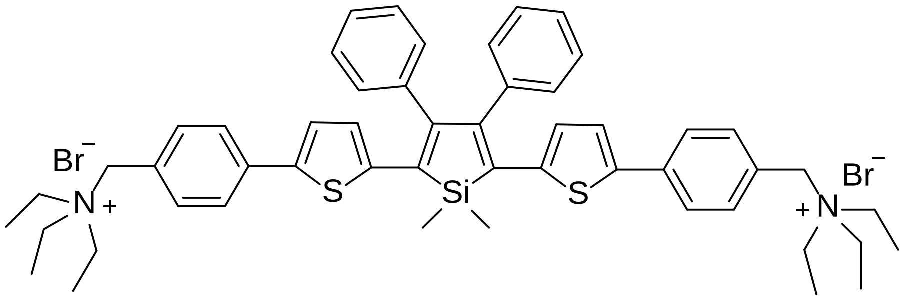

Molecular Formula of Silole-SNBr, MW=965.2569, $C_{52}H_{63}Br_2N_2S_2Si_2$.

Experimental details for Supplementary Figure 12

For all samples in Supplementary Figure 12, we used AAO membranes with larger diameter as 55 ± 15 nm for metal deposition and tests, which shows a good

performance of ionic gating according to the results in our previous work (*Nat. Commun.* **9**, 40 (2018)). The speeds of all depositions were set as 0.01 nm·s^{-1}, which is same with the deposition speed in the manuscript. The duration of Au deposition was prolonged from 1000 s (for Sample in Fig. 2 i and m) to 2000 s for the sample in Supplementary Fig.12 c and e, on purpose of extending the Au distribution in the nanochannels. Then, for the sample of Supplementary Fig.12 e, the outmost of the Au were further deposited by carbon at the speed of 0.01 nm·s^{-1} for 200 s. The samples used in Supplementary Fig.12 e and Supplementary Fig.13 underwent the same deposited processing. While, for the sample in Supplementary Fig.12 c, no further deposition took place on the outmost of the Au. For the samples in Supplementary Fig.12 a, the carbon deposition firstly took place at the outmost of the raw AAO membrane at the speed of 0.01 nm·s^{-1} for 2000 s. Then, an Au film was deposited on the outmost of the carbon at the speed of 0.01 nm·s^{-1} for 200 s. The Au region for the three groups of membranes were modified by capture probe DNA through Au-thiol bonding and then forming supersandwich DNA structures, according to the same procedure in the manuscript. The I-V tests were performed in 0.1 M KCl (pH=7.0).

For ATP detection, the capture-probe-modified AAO membranes (Supplementary Fig.12 e) were first put into a Tris buffer solution containing signal probe and DNA linker (1 μM each) for 12 h to allow sufficient assembly of DNA nanostructures. The resulting membrane were immersed in Tris buffer solution with different concentrations of ATP (from 1 nM to 1 mM) for 3 h before electrical measurements. ATP sensing was also performed in a tris-buffer contains NTPs (CTP, GTP or UTP of 1 μm for each), respectively.

Supplementary Figures

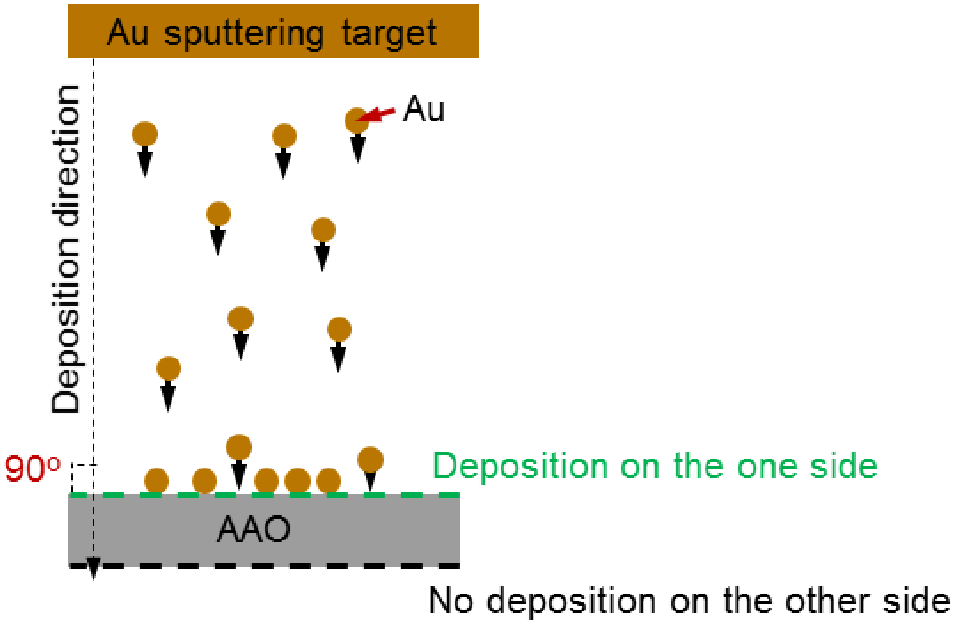

Supplementary Figure 1 | Sketch map of metallic depositions. The Au (or ITO) deposition perpendicular to the outmost of AAO and at the one side of the AAO membrane's OS.

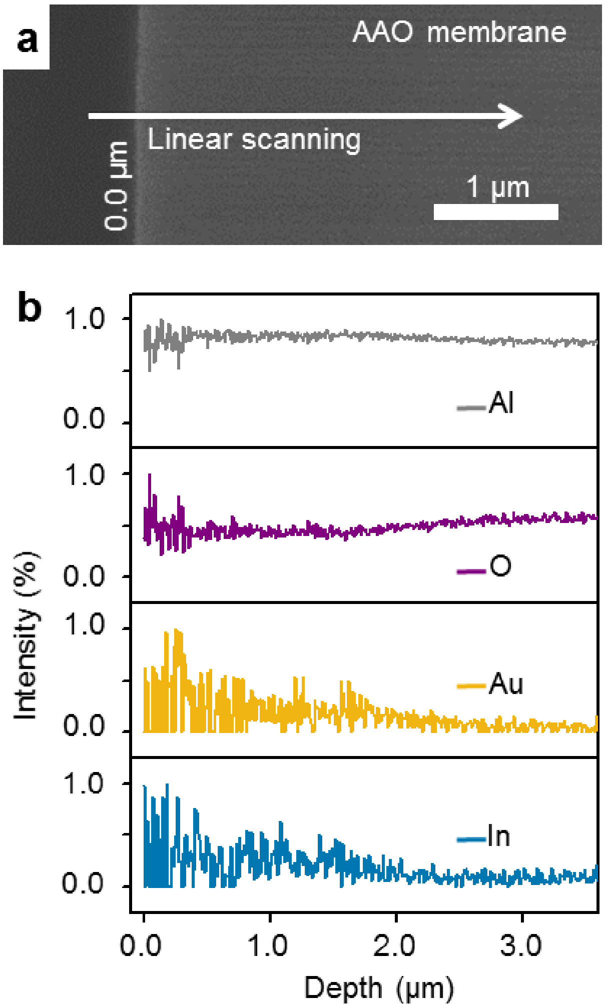

Supplementary Figure 2 | Linear scanning of EDS along the nanochannels. (a) SEM image of the tip of the AAO membrane. Linear scanning taking along the channel. (b) An AAO membrane with the perpendicular deposition of firstly Au through 100 s and secondly ITO through 100 s.

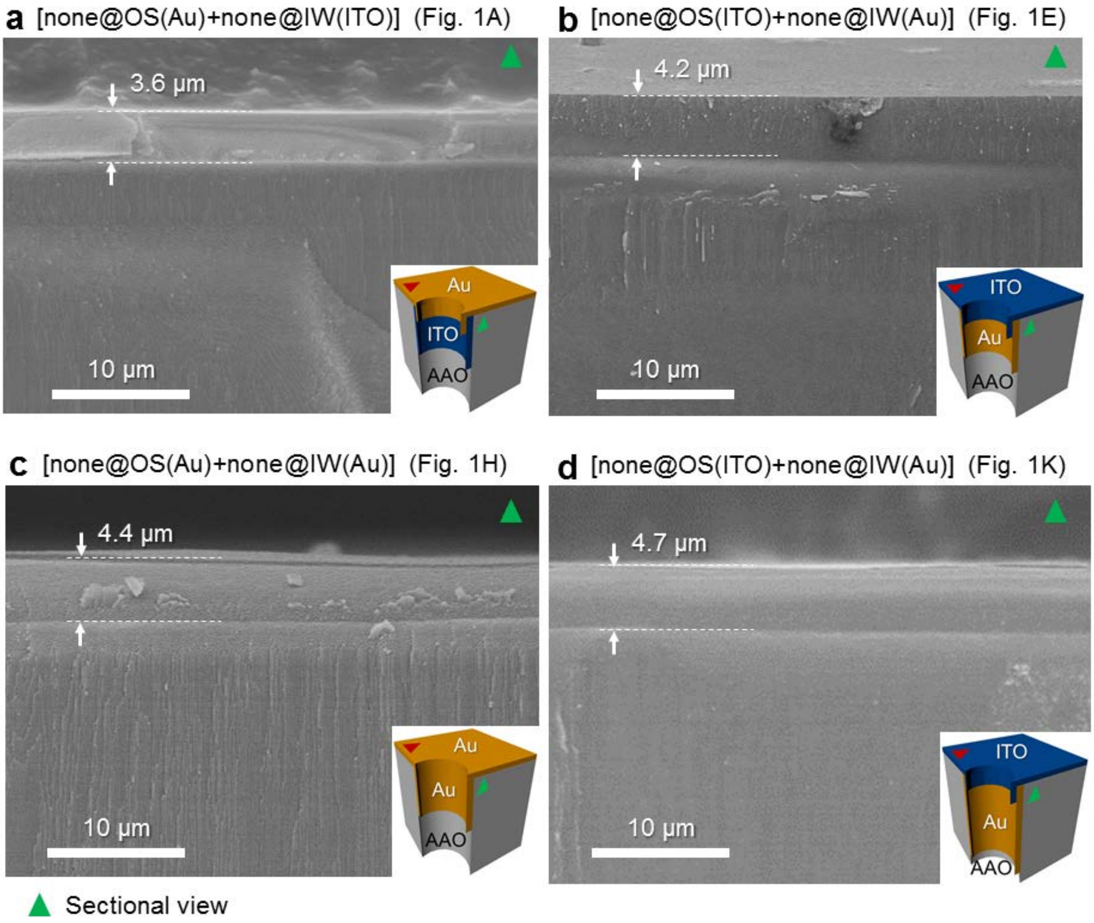

Supplementary Figure 3 | The sectional view of the membrane after TOF-SIMS. A square hole is dug by TOF-SIMS. SEM images record the sectional view of the holes. The intensity records of TOF-SIMS end at the bottom of the holes. (a) Fig. 2a, (b) Fig. 2e, (c) Fig. 2i and (d) Fig. 2m.

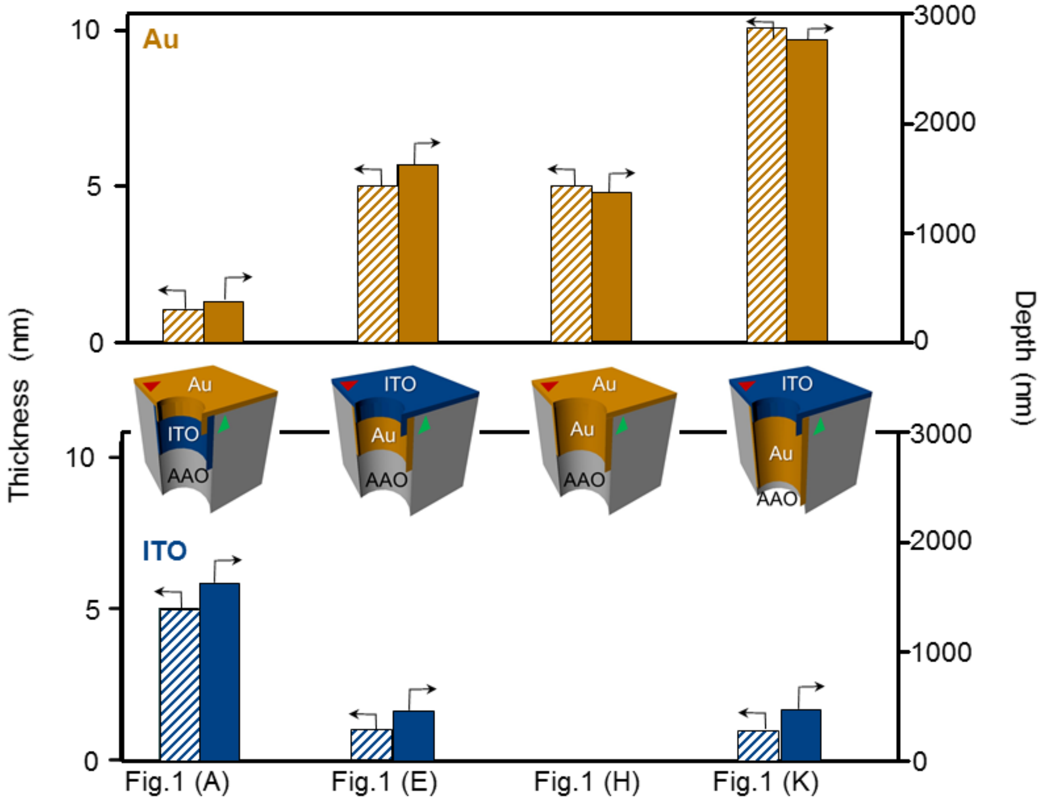

Supplementary Figure 4 | Thickness and the depth of the deposited elements. Left bars reflect the thickness according to the built-in parameter of the instrument, which is calibrated by the deposited thickness on the flat silicon wafer at nanometer level. Right bars reflect the depth of deposited element tested by TOF-SIMS. Au (top Figure) or ITO (bottom Figure). All samples are from Fig. 2.

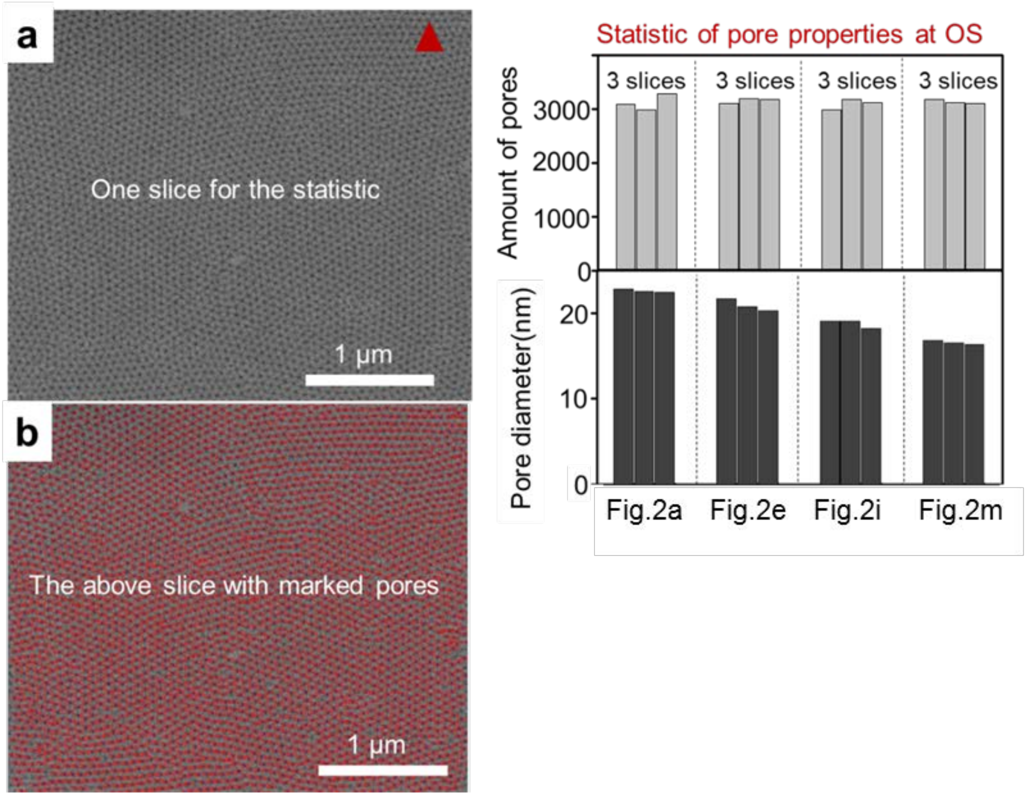

Supplementary Figure 5 | Statistic of geometrical parameter of the membranes. (a) A slice of SEM image measured from vertical view and (b) all pores in (a) are labelled, 3109 pores for the slice. (b) Statistic of amount (top) and pore size (bottom) for Fig. 2a, Fig. 2e, Fig. 2i and Fig. 2m. Statistic at around 9000 pores (three slices) for each kind of the deposited AAO membrane.

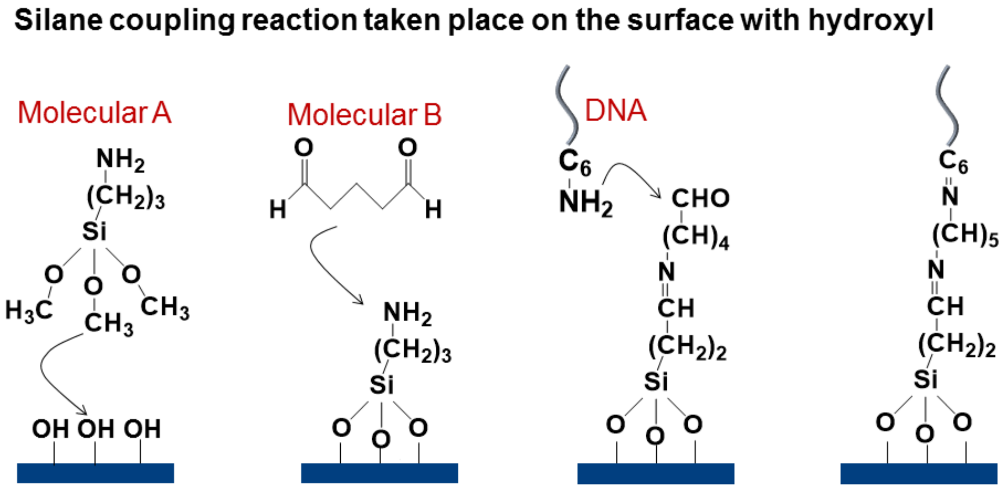

Supplementary Figure 6 | Sketch map of the silane coupling reaction. Molecular A: (3-aminopropyl)triethoxysilane (APTES); Molecular B: glutaraldehyde.

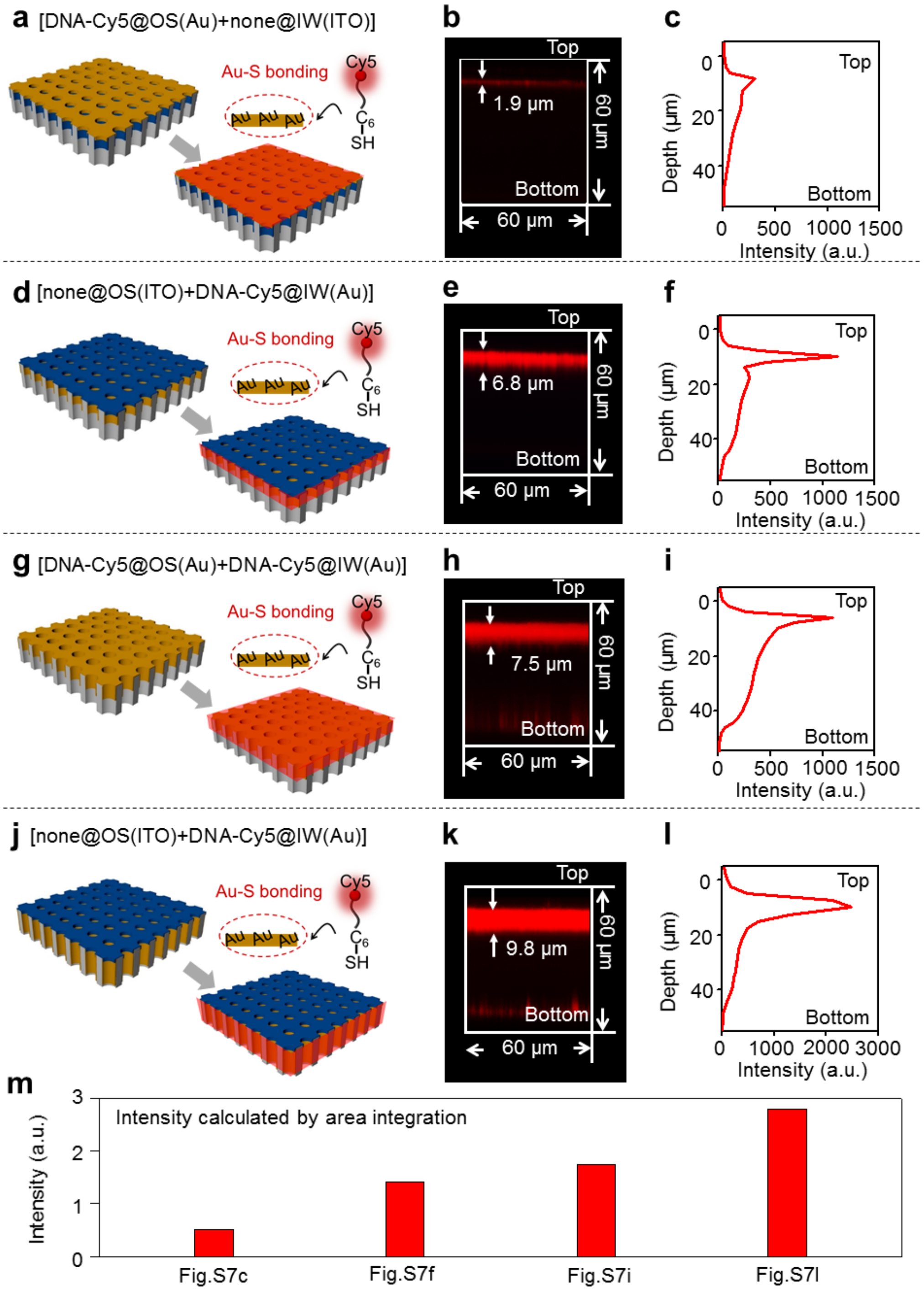

Supplementary Figure 7 | The LSCM images of DNA-Cy5 along nanochannels. Simple DNA-Cy5 (-SH modified at the 3' end) is added in the metallic deposited membranes. (a, b, c) [DNA-Cy5@OS(Au)+ none@IW(ITO)], (d, e, f) [none@OS(ITO)+DNA-Cy5@IW(Au)], (g, h, i) [DNA-Cy5@OS(Au)+DNA-Cy5@IW(Au)] and (j, k, l) [none@OS(ITO)+DNA-Cy5@IW (Au)]. (m) The integrated intensity of DNA-Cy5 along nanochannels calculated from the LSCM data (c, f, i, l).

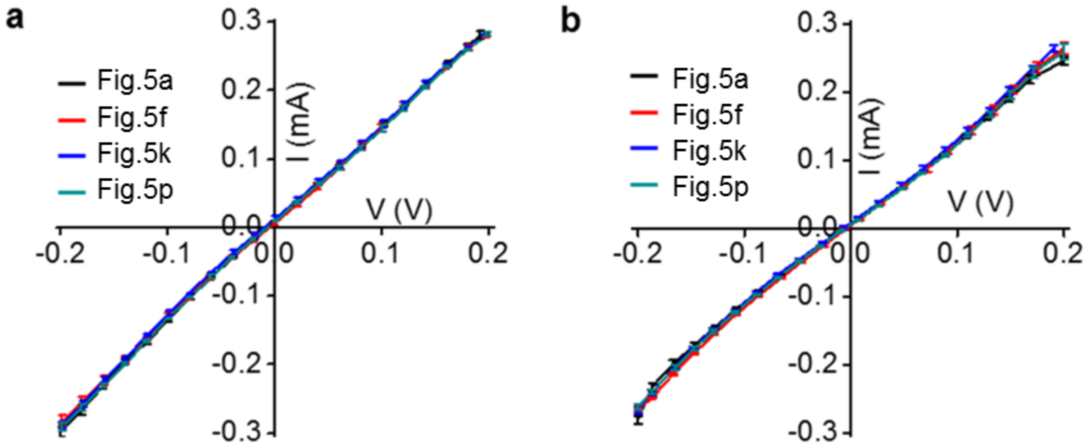

Supplementary Figure 8 | The I-V curves after the metallic depositions. The different process of metallic depositions at the step of none-FE (Fig. 5). (a) the I-V curves of the series for the hybridization between two DNA stands. (b) the I-V curves of the series for the supersandwich DNA structures. Error bars represent standard deviations of the measured samples. Five experimental replicates for each data.

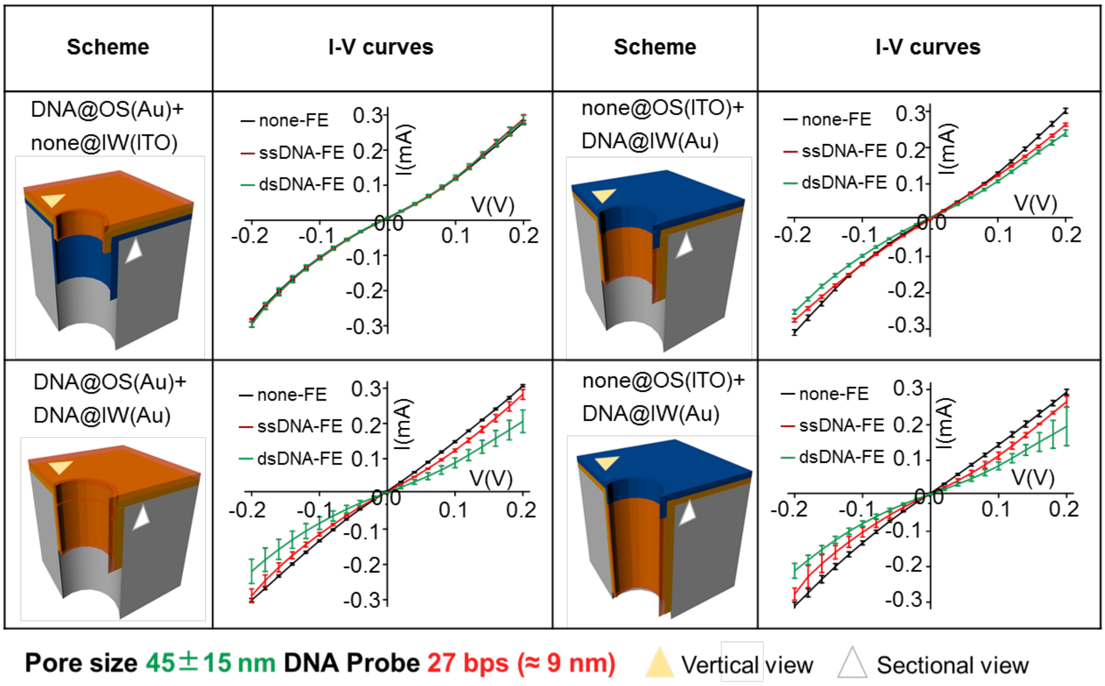

Supplementary Figure 9 | I-V curves of the membrane with pore size (55 ± 15 nm). The I-V curves are tested after DNA grafting and hybridization DNA with 27 bps (≈ 9 nm). Four samples with the same varied stacking orders of Au and ITO as Fig. 2, which are [DNA @OS(Au)+none@IW(ITO)], [none@OS(ITO)+DNA@IW(Au)], [DNA@OS(Au)+ DNA@IW(Au)] and [none@OS(ITO)+DNA@IW(Au)] by atomic layer deposition (ALD). I-V curves are listed to characterize the current of the state as: none-FE grafting, ssDNA-FE grafting and dsDNA-FE grafting in four samples. Error bars represent standard deviations of the measured samples. Five experimental replicates for each data.

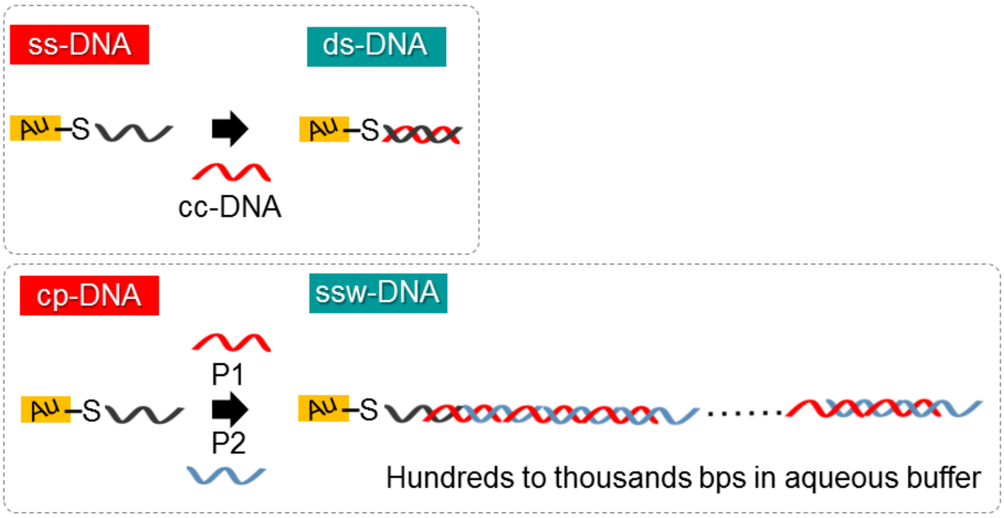

Supplementary Figure 10 | Sketch map of the DNA hybridization. Formation process from ss-DNA to ds-DNA or from cp-DNA to ssw-DNA.

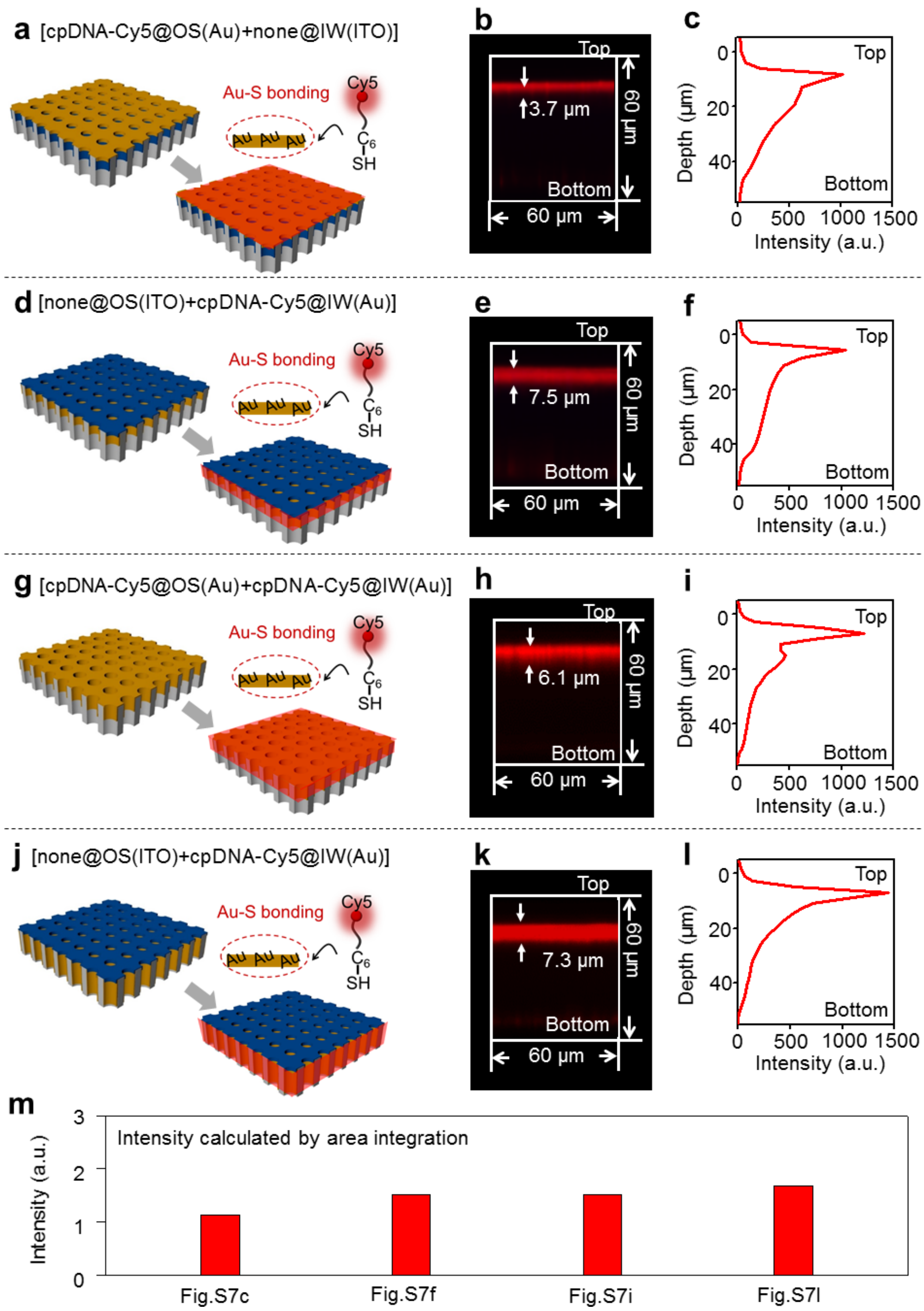

Supplementary Figure 11 | The LSCM images of cpDNA-Cy5 along nanochannels. Simple cpDNA-Cy5 (-SH modified at the 3' end) is added in the metallic deposited membranes. cpDNA-Cy5 is the capture probe of the DNA supersandwich structure. (a, b, c) [cpDNA-Cy5@OS (Au)+none@IW(ITO)], (d, e, f) [none@OS(ITO)+cpDNA-Cy5@IW(Au)], (g, h, i) [cpDNA-Cy5@OS(Au)+cpDNA-Cy5@IW(Au)] and (j, k, l) [none@OS(ITO)+DNA-Cy5@IW(Au)]. (m) The integrated intensity of DNA-Cy5 along nanochannels calculated from the LSCM data (c, f, i, l).

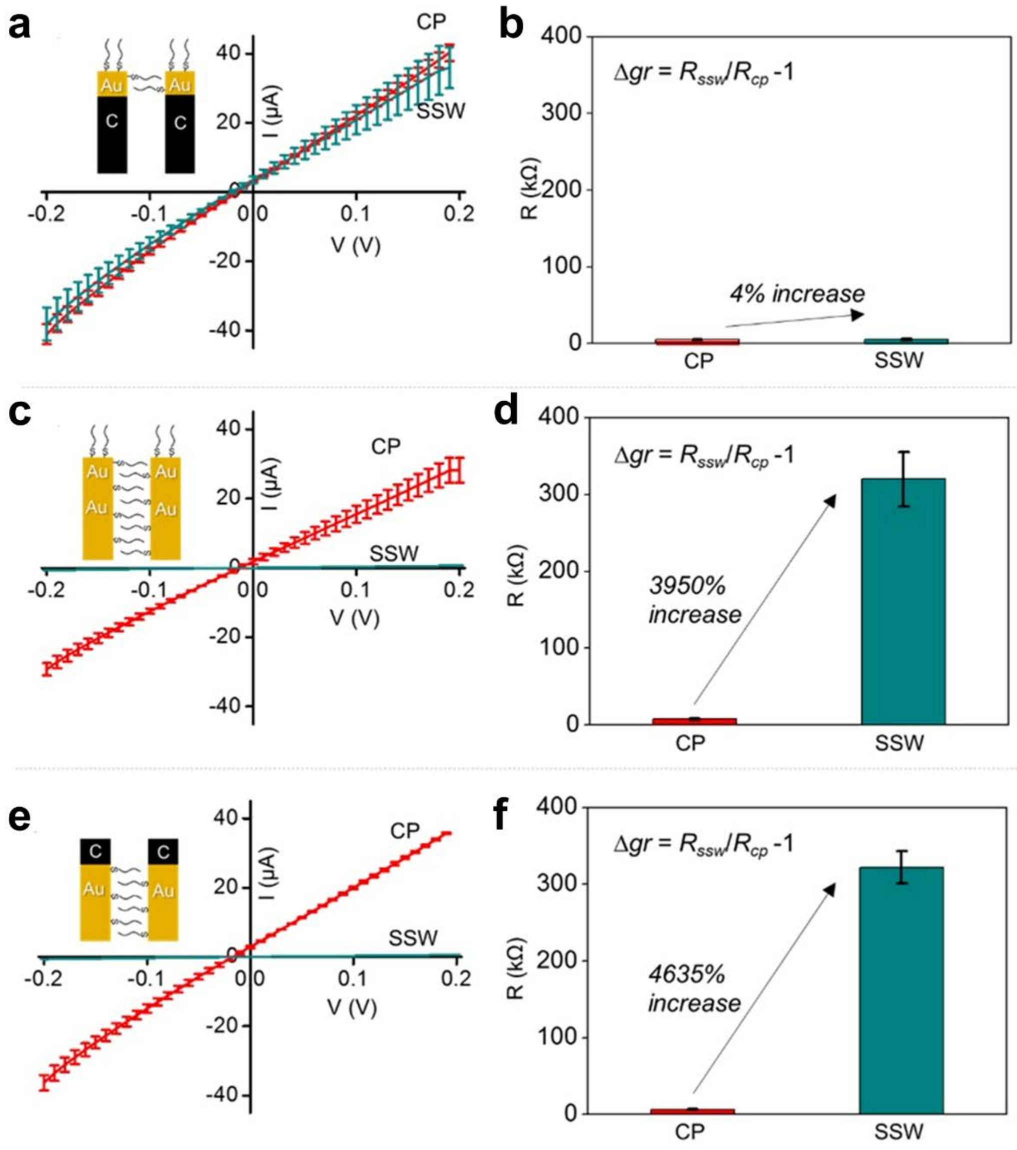

Supplementary Figure 12 | I-V curves of the membrane modified with ssw-DNA. Left Figures: The I-V curves of three kinds of membranes modified with capture probe (red line) and after modified with supersandwich DNA structure (green line). (a) The I-V curves of [sswDNA@OS(Au)+none@OS(C)], (c) The I-V curves of [sswDNA @OS(Au)+sswDNA@OS(Au)], (e) The I-V curves of [none@OS(C)+sswDNA@OS (Au)]. CP: nanochannels modified with the capture probe of supersandwich DNA structure, SSW: nanochannels modified with the supersandwich DNA structure. The corresponding resistance calculated from the I-V curves (b, d and f). Experiments detailed in Supplementary Methods 1.2. Error bars represent standard deviations of the measured samples. Five experimental replicates for each data.

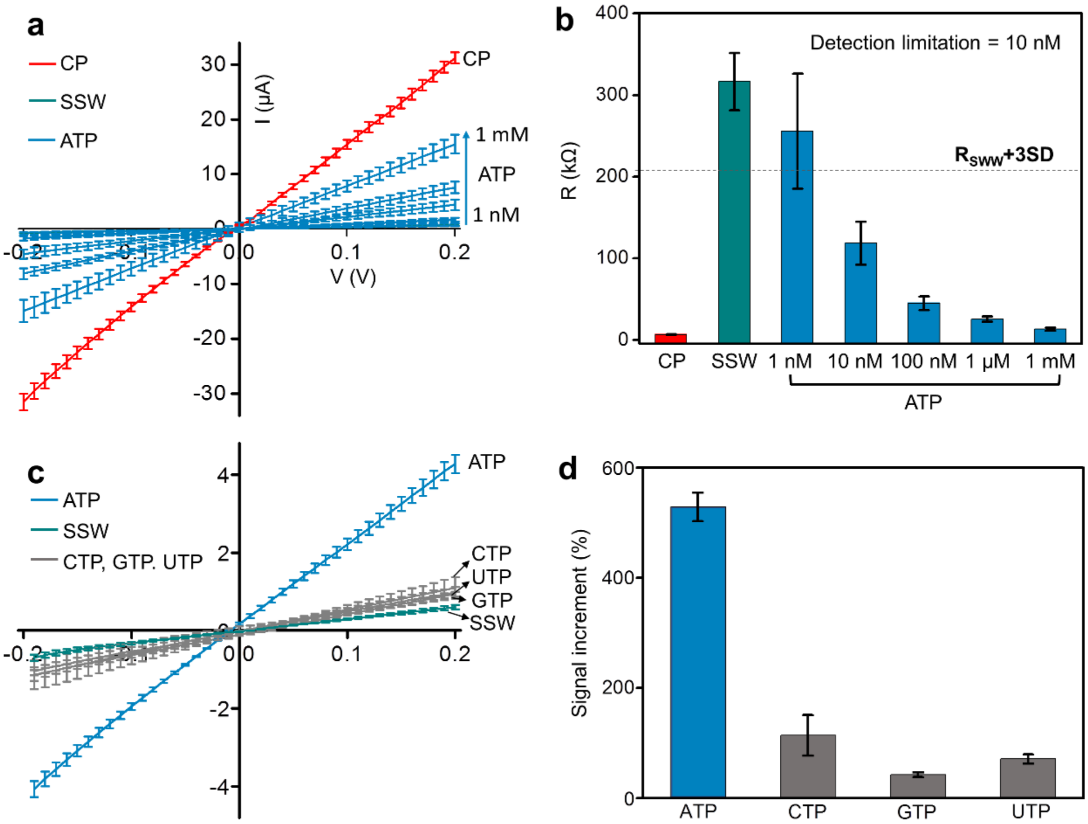

Supplementary Figure 13 | Detection of ATP by using the nanochannels. The nanochannels were modified by supersandwich DNA structures. (a) I-V curves before and after ATP treatment with different concentration from 1 nM to 1mM. CP: nanochannels modified with the capture probe of ssw-DNA structure, SSW: nanochannels modified with the ssw-DNA structure. (b) Dose-response curves. The signal increase is defined as $(I_{ATP}-I_{SSW})/I_{SSW}$, where I mean the current at 2.0 V. (c) Our system is highly selective for ATP, in contrast with other three types of nucleoside triphosphate (NTP), CTP, GTP, and UTP. (d) The signal increment of the I-V curves in Supplementary Fig. 13 c. The ATP will trigger the disassembly of supersandwich DNA structure (ssw-DNA) and further leads to the rise of transmembrane ionic current. The variation of ionic currents can be used for ATP detections. The detection of ATP has been achieved by using our system with a sensitivity to 10 nM. Meanwhile, our system shows higher selectivity towards its target (ATP) than the other three types of NTPs. Experiments detailed in Supplementary Methods 1.2. Error bars represent standard deviations of the measured samples. Five experimental replicates for each data.

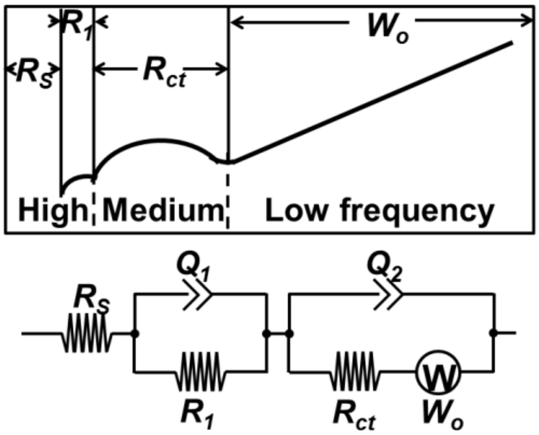

Supplementary Figure 14 | EIS sketch and equivalent circuit. Top Figure is the EIS sketch labelling the impedance elements in different frequency, Bottom Figure is the equivalent circuit used to simulate the impedance spectra. (*Adv. Mater.* **2016**, *28*, 460.)

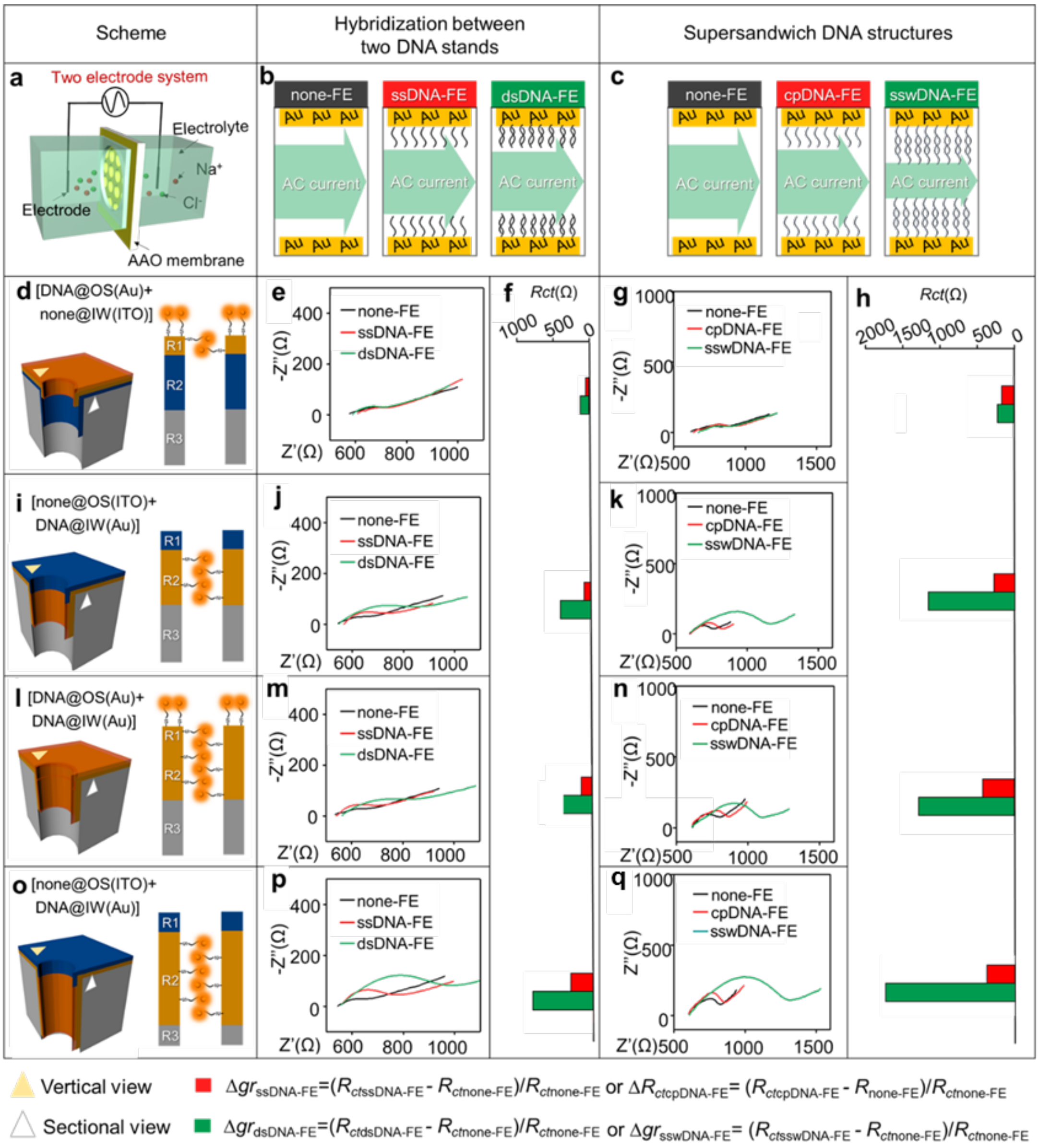

Supplementary Figure 15 | Gating efficiency of FE_{OS} and FE_{IW} by EIS. The EIS are applied for characterizing the resistance of membranes, reflecting the gating efficiency of the solid-state membrane after introducing single-stand and double-stand DNAs. The pore size the AAO membranes is 25±5 nm and the pore depth is around 40 µm. (a) the cartoon of two-electrode system used in electrochemical characterizations. (b) Three states of DNAs at Au surface as no DNA grafting (none-FE), after single-stand DNA grafting (ssDNA-FE) and after the hybridizations between ss-DNAs and their complete complementary cc-DNAs (dsDNA-FE). (c) A DNA supersandwich structure (ssw-DNA). Three states of DNAs at Au surface as no DNA grafting (none-FE), after single-stand capture probe grafting (cpDNA-FE) and supersandwich structure DNA grafting (sswDNA-FE) (d, i, l and o). EIS curves characterize the current of the state as: none-FE grafting, ssDNA-FE grafting and

dsDNA-FE grafting in four samples which are described in (e, j, m and p); none-FE grafting, cpDNA-FE grafting and sswDNA-FE grafting in four samples which are described in (g, k, n and q). Varied Δgr are defined in the bottom part. Calculated Δgr are shown in (f) for DNA hybridization and (h) for sswDNA formation. Error bars represent standard deviations of the measured samples.

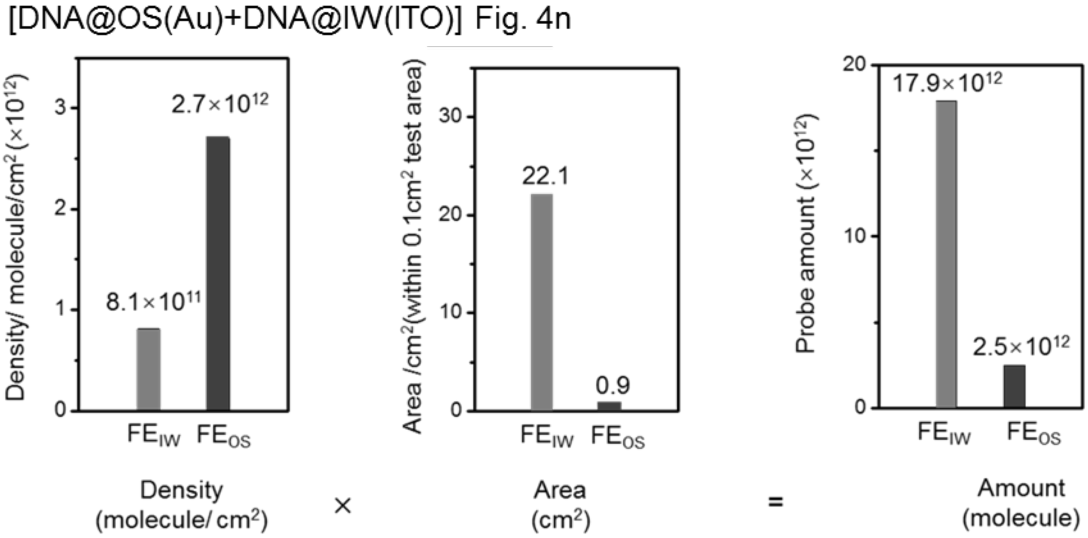

Supplementary Figure 16 | Comparison of DNA amount at different domains. Comparison of assembly density, regionally functional area, and probe molecule amount at different domains of the nanochannels (for the sample in Fig. 4n).

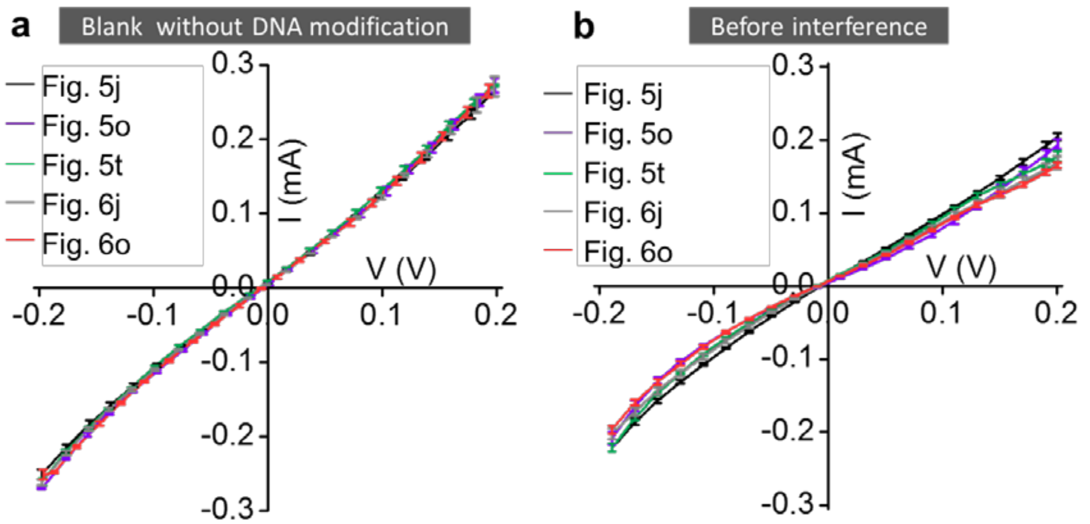

Supplementary Figure 17 | I-V curves of the superficially functionalized membranes. (a) Blank membrane without DNA modification and (b) Functional membrane with DNA modification but before interference by charged molecules (Fig. 5 and Fig. 6). Error bars represent standard deviations of the measured samples. Five experimental replicates for each data.

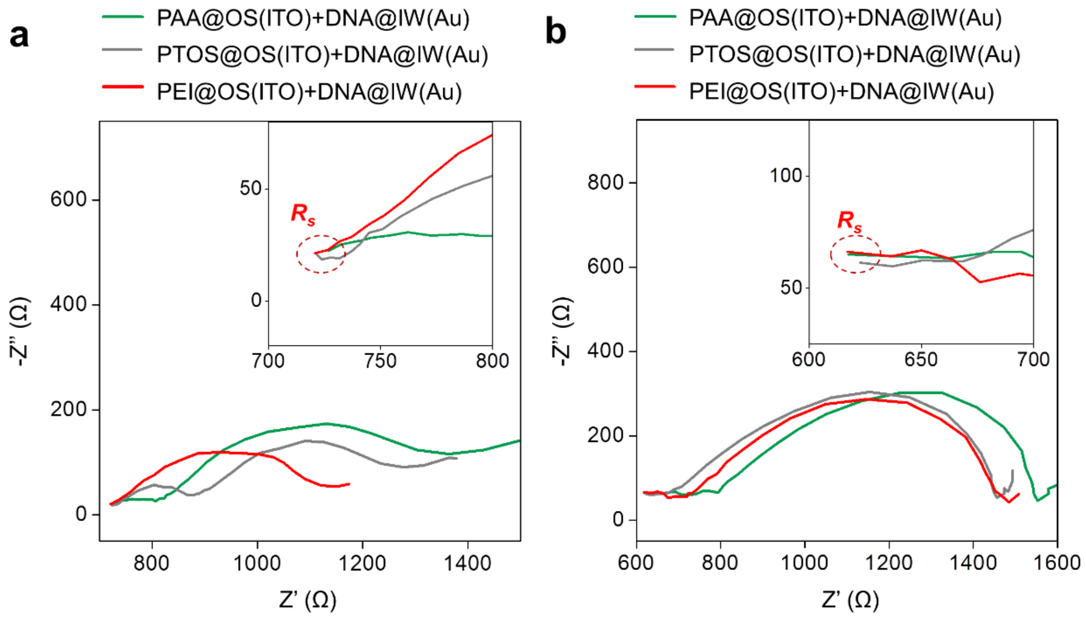

Supplementary Figure 18 | The EIS spectra of the membranes in Fig. 5 and Fig. 6. [Grey line: PTOS@OS(ITO)+ DNA@IW(Au), Green line: PAA@OS(ITO)+DNA@IW(Au) and Red line: PEI@OS(ITO)+DNA@IW(Au)] (a) at 0 V and (b) at 2 V, respectively. The enlarge Figure is to show that the solution resistance (R_s) of three membranes is approximately equal.

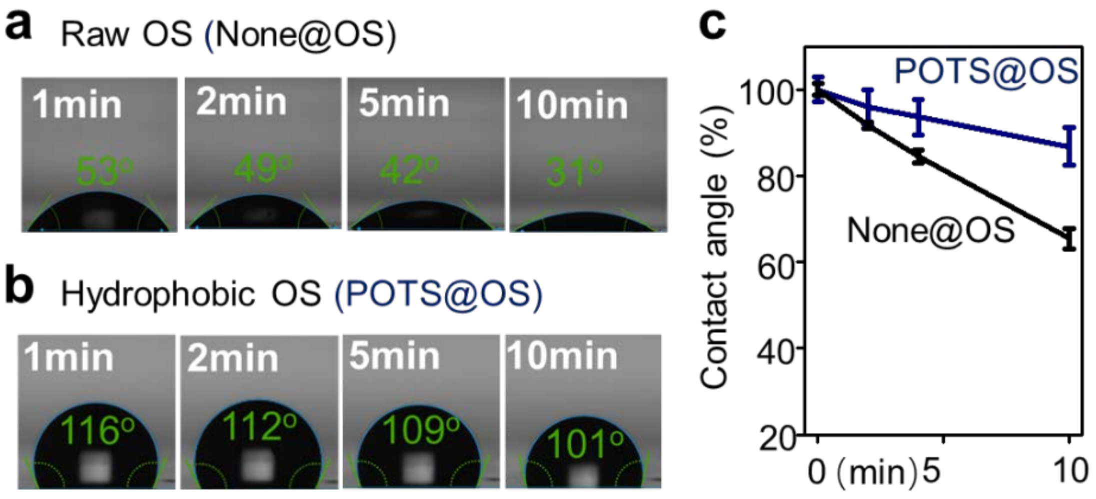

Supplementary Figure 19 | Contact angle tests of the functionalized membranes. The contact angle (CA) varies with the duration due to the capillary force of nanochannels. Four durations are recorded as 1 min, 2 min, 5 min and 10 min. (a) the contact angle images of aqueous DNA solution (1 μM) at the raw OS [None@OS(ITO)+DNA@IW(Au)] after different duration. (b) the contact angle images of aqueous DNA solution (1 μM) at the hydrophobic OS [PTOS@OS(ITO)+DNA@IW(Au)] after different duration. (c) the variation of contact angles vs durations of the aqueous DNA droplet at the raw and hydrophobic OS. Error bars represent standard deviations of the measured samples. Five experimental replicates for each data.

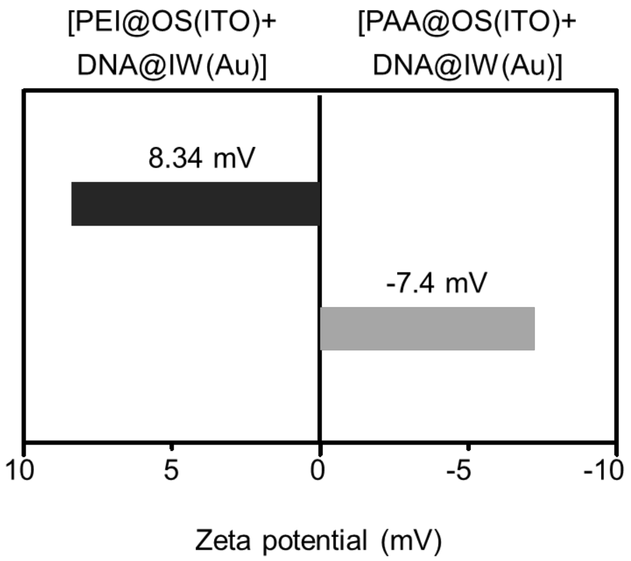

Supplementary Figure 20 | Zeta potential of PEI or PAA coating membrane. The zeta potential of the PEI coating membrane is collected at the pH ≈ 7.2, while the potential of the PAA coating membrane was collected at the pH ≈ 6.9.

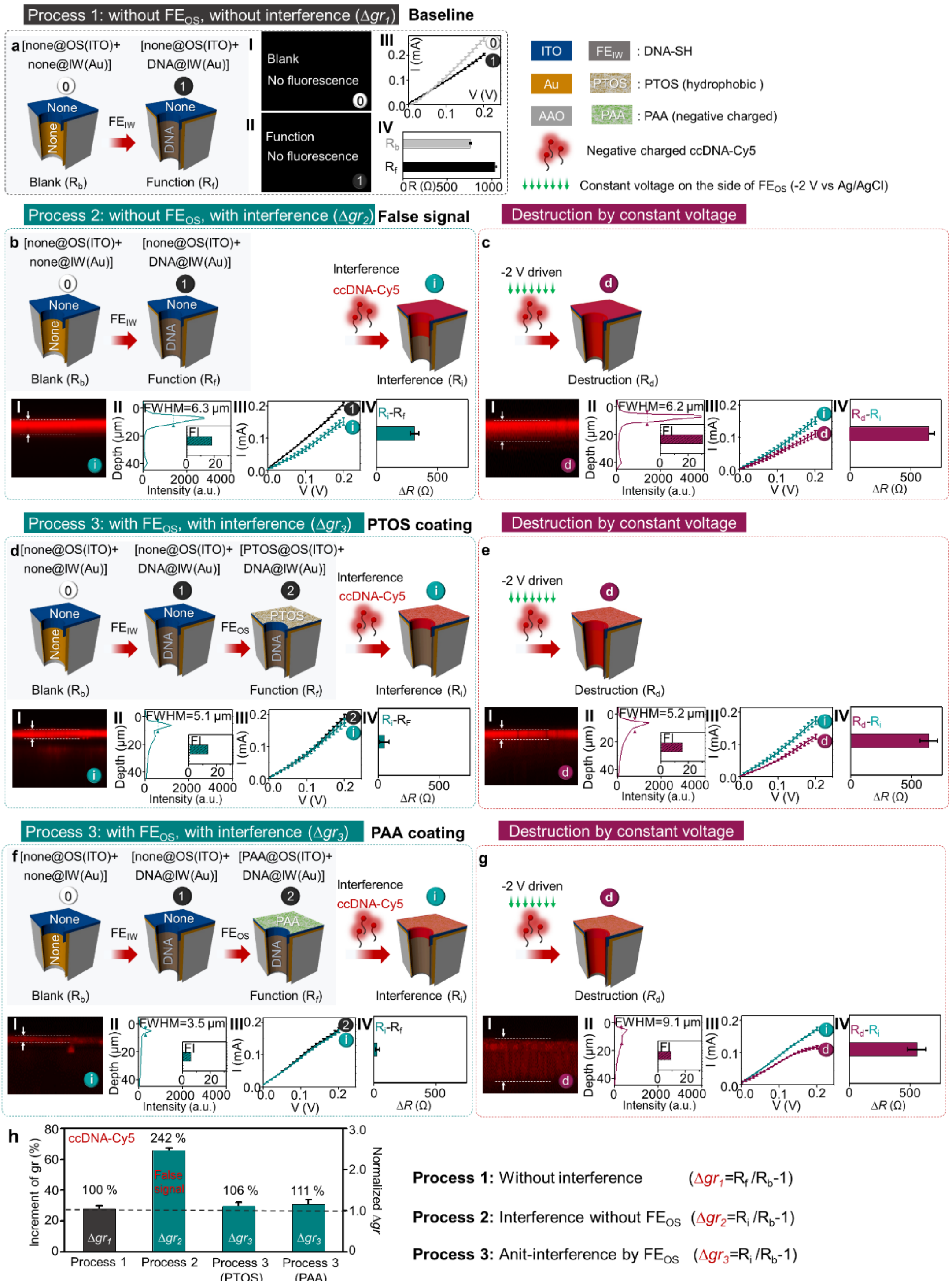

Supplementary Figure 21 | The chosen FE_{OS} with the anti-interference as Fig. 5. Three different processes (**Process 1** (**a**), **2** (**b**) and **3** (**d**/**f**)) are conducted to evaluate whether the FE_{OS}, we chosen, bring OS with the anti-interference ability. (h) Comparison of the Δgr from the three processes mentioned above, and the following is the definition for them: Δgr_1 = R_f /R_b-1(for **Process 1**); Δgr_2 = R_i /R_b-1(for **Process 2**); Δgr_3=R_i /R_b-1 (for **Process 3**). Error bars represent standard deviations of the measured samples. Five experimental replicates for each data.

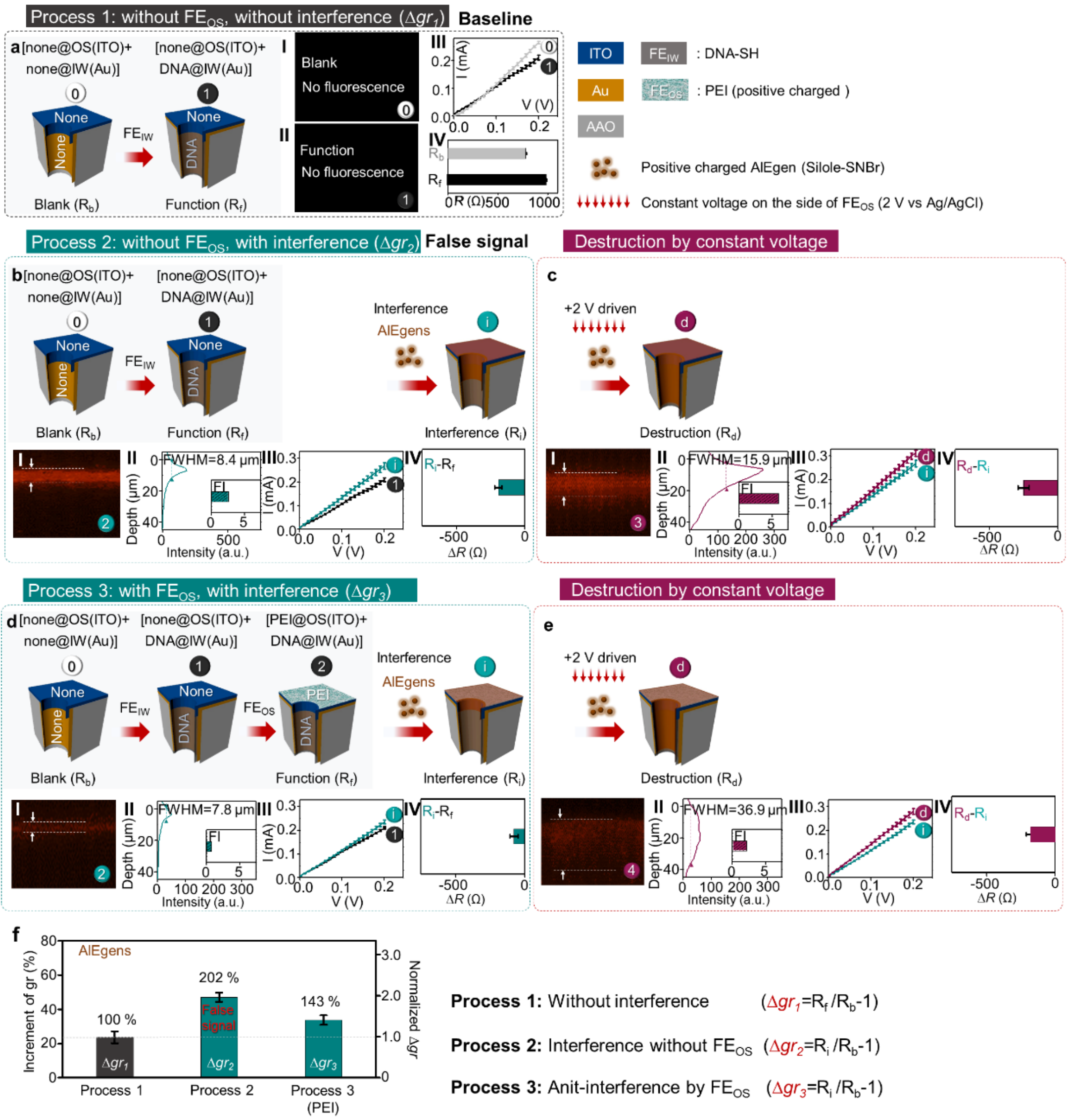

Supplementary Figure 22 | The chosen FE_{OS} with the anti-interference as Fig. 6. Three different processes (**Process 1** (**a**), **2** (**f**) and **3** (**d**)) are conducted to evaluate whether the FE_{OS}, we chosen, bring OS with the anti-interference ability. AIEgen is chosen as the interference molecule. (**f**) Comparison of the Δgr from the three processes mentioned above, and the following is the definition for them: $\Delta gr_1 = R_f / R_b$-1 (for **Process 1**); $\Delta gr_2 = R_i / R_b$-1(for **Process 2**); $\Delta gr_3 = R_i / R_b$-1(for **Process 3**). Error bars represent standard deviations of the measured samples. Five experimental replicates for each data.

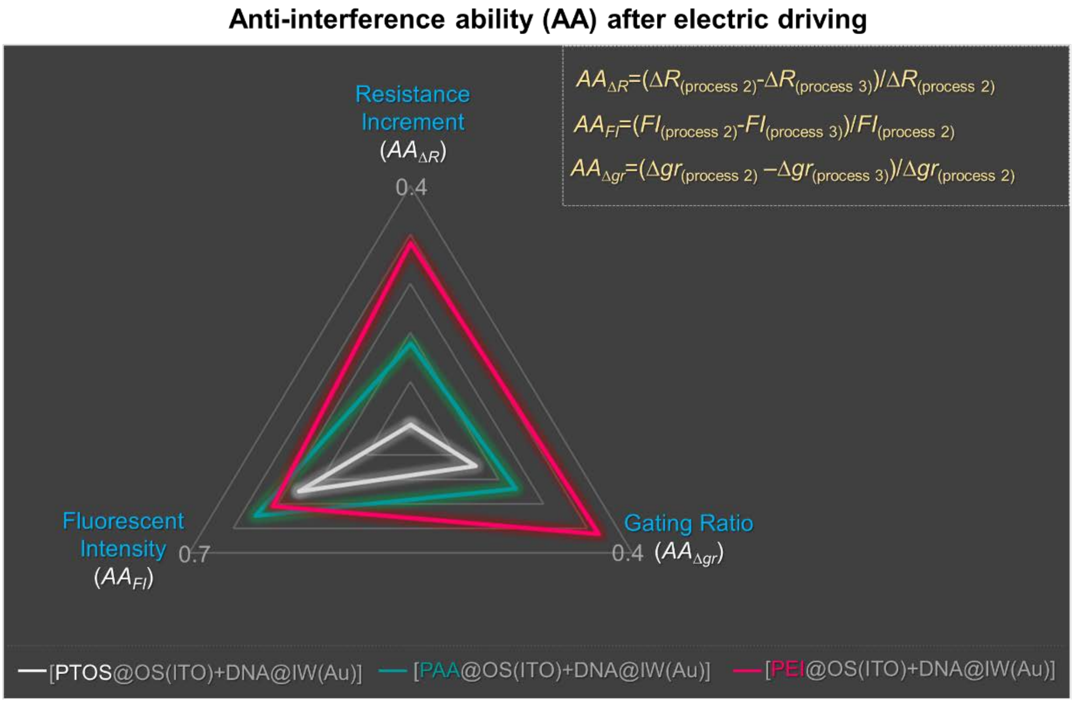

Supplementary Figure 23 | The radar map of anti-interference after 2 V driving. The three parameters are considered as the resistance variation from step i to step d ($AA_{\Delta R}$), gating ratio ($AA_{\Delta gr}$), fluorescent intensity (AA_{FI}). The calculated formulas are listed as $AA_{\Delta R}$ = (ΔR(process 2)-ΔR(process 3))/ΔR(process 2), AA_{FI}=(FI(process 2)-FI(process 3))/FI(process 2), $AA_{\Delta gr}$=(Δgr(process 2) – Δgr(process 3))/ Δgr(process 2) (where the Δgr are calculated by taking R_{Blank} as 100 %). The bigger area of the polygon, and the greater anti-interference of FE_{OS}.

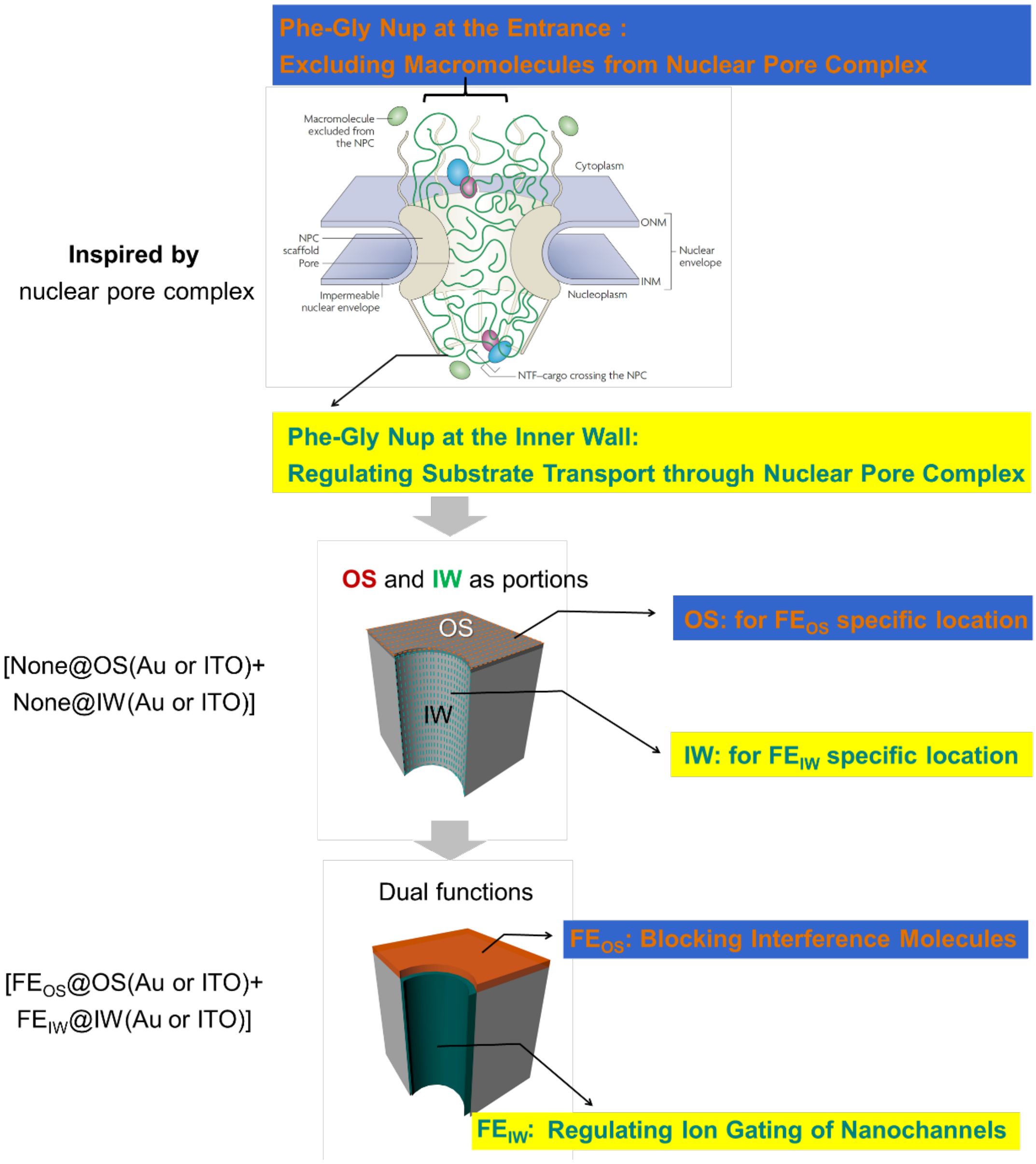

Supplementary Figure 24 | Analogical function divisions in a nuclear pore complex. In a nuclear pore complex, the Phe-Gly Nup meshwork at the entrance of nuclear pore complex (analogous to FE_{OS}) physically excludes macromolecule from the nuclear pore complex. Meanwhile, the Phe-Gly Nups inside nuclear pore complex (analogous to FE_{IW}) regulate the transport of substrates through nuclear pore complex. Produced with permission from *Nat. Rev. Mol. Cell. Bio.* **11**, 491-501(2010), Copyright [2010], Springer Nature. All right reversed.

Supplementary Tables

Supplementary Table 1 | Names of used samples

Name	Element		Regional modified FEs	
	OS	IW	FEs@OS	FEs@IW
Fig. 2a	Au	ITO	None	None
Fig. 2e	ITO	Au	None	None
Fig. 2h	Au	Au	None	None
Fig. 2k	ITO	Au	None	None
Fig. 3b	Au	ITO	DNA-Cy5	DNA-FITC
Fig. 3f	ITO	Au	DNA-FITC	DNA-Cy5
Fig. 3i	Au	Au	DNA-Cy5	DNA-Cy5
Fig. 3l	ITO	Au	DNA-FITC	DNA-Cy5
Fig. 4d	Au	ITO	DNA-Cy5	None
Fig. 4i	ITO	Au	None	DNA-Cy5
Fig. 4l	Au	Au	DNA-Cy5	DNA-Cy5
Fig. 4o	ITO	Au	None	DNA-Cy5
Fig. 4g	Au	ITO	cpDNA-Cy5	None
Fig. 4k	ITO	Au	None	cpDNA-Cy5
Fig. 4n	Au	Au	cpDNA-Cy5	cpDNA-Cy5
Fig. 4q	ITO	Au	None	cpDNA-Cy5
Fig. 5f<br>Fig. 6f	Au	ITO	None	DNA-SH
Fig. 5k	ITO	Au	PTOS	DNA-SH
Fig. 5p	Au	Au	PAA	DNA-SH
Fig. 6k	ITO	Au	PEI	DNA-SH

Supplementary Table 2 | Abbreviations

No	Abbreviation	Full name
1	FE	Functional element
2	IW	Inner wall
3	OS	Outer surface
4	FE_{IW}	FEs at the inner wall
5	FE_{OS}	FEs at the outer surface
6	TOF-SIMS	Time of flight secondary ion mass spectrometry
7	FITC	Fluoresceine isthiocyanate
8	Cy5	Cyaine-5
9	LSCM	Laser scanning confocal microscopes
10	Δgr	Increment of gating ratio
11	ss-DNA	Single-strand DNA
12	ds-DNA	Double-strand DNA
13	cp-DNA	DNA capture probe of supersandwich DNA structure
14	ssw-DNA	Supersandwich DNA structure
15	cc-DNA	Complete complementary DNA
16	EIS	Electrochemical impedance spectroscopy
17	POTS	Perfluorooctyltriethoxy silane
18	PAA	Polyacrylic acid
19	PEI	Polyetherimide
20	AA	Anti-interference ability

Supplementary Table 3 | DNA sequences

	Sequence	5' end	3' end
DNA	Sequence 1	no	no
DNA-SH	Sequence 1	no	-SH
DNA-NH	Sequence 1	no	-NH
DNA-FITC	Sequence 1	FITC	-NH
DNA-Cy5	Sequence 1	Cy5	-SH
ccDNA	Sequence 2	no	no
ccDNA-Cy5	Sequence 2	Cy5	no
cpDNA-SH	Sequence 3	no	-SH
cpDNA-Cy5	Sequence 3	Cy5	-SH
P1	Sequence 4	no	no
P2	Sequence 3	no	no

	From 5' end to 3' end
Sequence 1	TTTTATAAGTTTGGTGGTTAGAGAGTG
Sequence 2	CACTCTCTAACCACCAAACTTATAAAA
Sequence 3	CGGCACCTGGGGGAGTATTGCGGAGGAAGGTGCCG
Sequence 4	TACTCCCCCAGGTGCCGACGGCACCTTCCTCCGCA

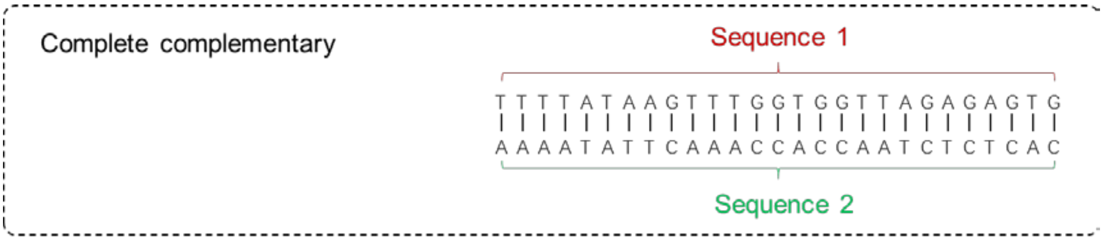

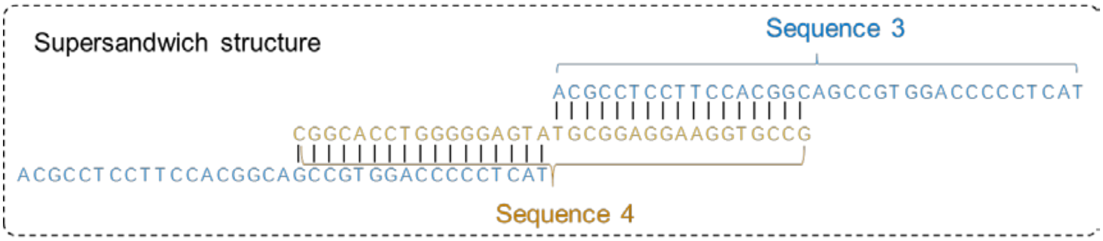

Supplementary Discussion

In the manuscript, we find that the fluorescent depth of DNA-Cy5 is far greater than the depth of the deposited Au (Fig. 2, 3). We assign the large disparities to the three probable reasons as followed:

There are the great disparities for the measuring accuracy between TOF-SIMS and LSCM.

Limited to the stepping motor, the measuring accuracy of LSCM is limited to 200 nm at the Z-axil. In the present work, we operate the Z-axil scanning of LSCM at the step of 500 nm with the consideration of the measuring accuracy and the possible fluorescence quenching. The measuring accuracy, however, of TOF-SIMS is 10 nm.

The data for the depth of Au from fluorescence is more than the actual value.

Even after washing with buffer, some of the DNA labeled with fluorescent remains in the nanochannels. We have tried two different fluorescent modification methods: (1) Two DNA sequences labeled with Cy5 and FITC, respectively, are acted as FEs in Fig. 3; (2) while DNA sequences labeled with only Cy5 are acted as FEs in Supplementary Fig. 7 and Supplementary Fig. 11. If we use the Data in (2) for Fig. 3e, i, m and q the disparity would be reduced, which may be due to the DNA in (2) remaining less in the nanochannels compared with the one in (1).

The Data for the depth of Au from TOF-SIMS is less than the actual value.

For the TOF-SIMS, we end the data collection at the end of a plateau of intensity which occurred after a sharp decline and remained for a quite long time (as shown in Supplementary Fig. 25). In the manuscript, we define the intensity of the plateau as the 0 % of relative intensity in TOF-SIMS. However, for Au, the absolute intensity is not zero (marked for the four kinds of deposited membrane in Supplementary Figure 25), which means the existence of Au. For example, the absolute intensity of the plateau in Supplementary Figure 25 a is 1346. Furthermore, even for the data in Fig. 2, when taking 2 % of peak intensity as the end, the Au depth will be much longer than the situation by taking 5 % of peak intensity as the end. This means that the data for

the depth of Au from TOF-SIMS in Fig. 3 e, i, m and q is distinctively less than the actuate value.

As a result, for the three possible reasons above, the large disparities of depth exist between LSCM and TOF-SIMS. However, TOF-SIMS and LSCM show the same trend for the depth of Au (Fig. 3 e, i, m and q), which means the data from these support each other to some extent.

Supplementary Table 4 | Depth calculations by TOF-SIMS and LSCM

	OS(Au)+IW(ITO)	OS(ITO)+IW(Au) Au with a short range	OS(Au)+IW(Au) Au with a short range	OS(ITO)+IW(Au) Au with a long range
**LSCM** (DNA-Cy5 + DNA-FITC) Fig. 3	3.4 μm	10.7 μm	9.2 μm	14.8 μm
**LSCM** (DNA-Cy5) Fig. S7	1.9 μm	6.8 μm	7.5 μm	9.8 μm
**TOF-SIMS** 2% as the end Fig. 2	0.78 μm	3.01 μm	2.52 μm	3.83 μm
**TOF-SIMS** 5% as the end Fig. 2	0.38 μm	1.63 μm	1.37 μm	2.76 μm

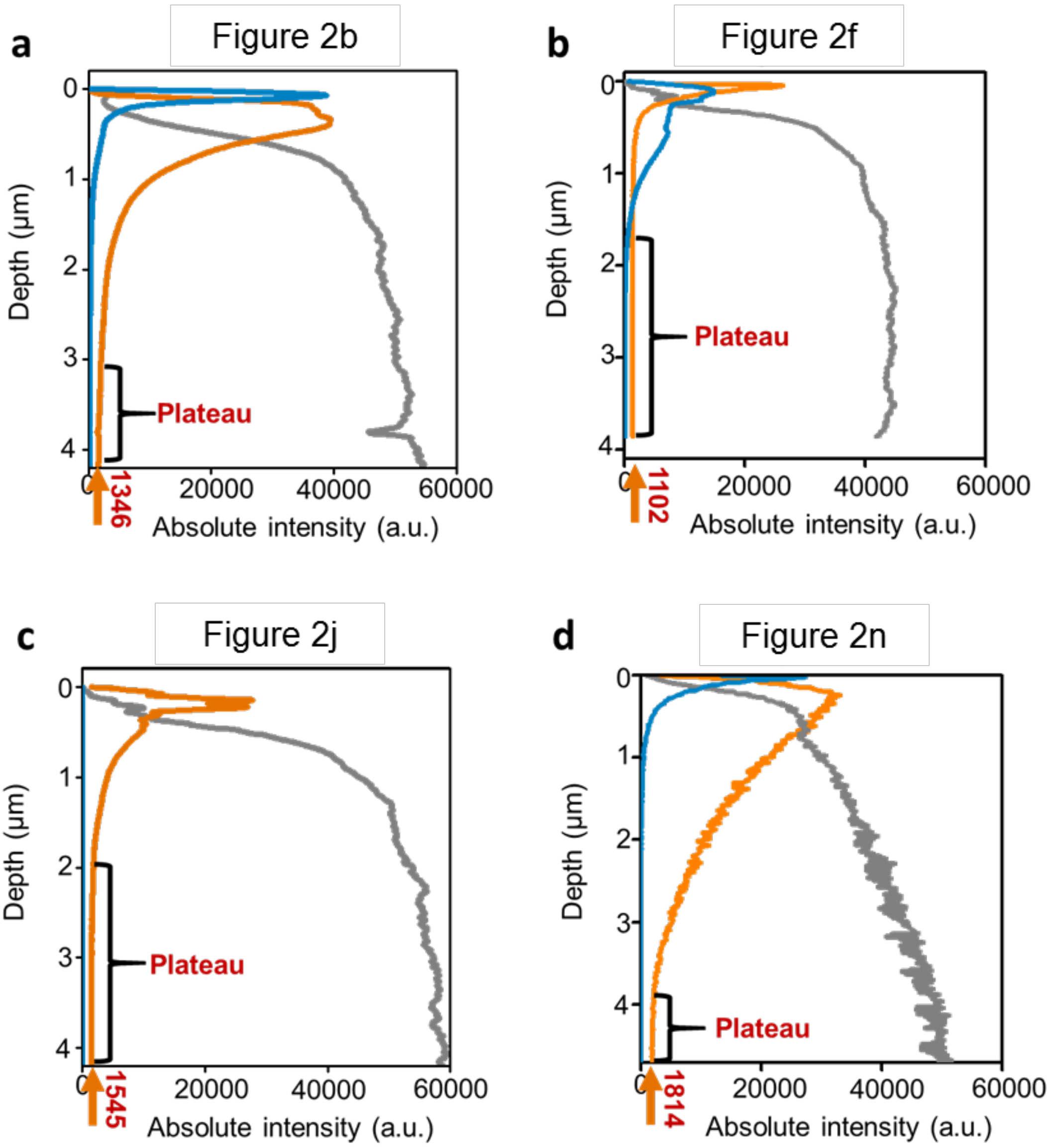

Supplementary Figure 25 | The profile of depth vs. intensity for TOF-SIMS. The intensity is converted to absolute intensity for Fig. 2: (a) Fig. 2b, (b) Fig. 2f, (c) Fig. 2j and (d) Fig. 2n.
